# Evaluation of efficacy and safety profile of tadalafil 5 mg daily dose in the tablet form versus oral dispersible film in men with mild-to-moderate erectile dysfunction: a comparative placebo-controlled study

**DOI:** 10.1007/s11255-024-04003-x

**Published:** 2024-03-04

**Authors:** Ahmad T. Motawi, Sameh Fayek GamalEl Din, Eslam M. Meatmed, Ibrahim Fahmy

**Affiliations:** Department of Andrology and STDs Kasr Al-Ainy, Sexual Medicine & STIs Department, Faculty of Medicine, Cairo University, Al-Saray Street, El Manial, Cairo, 11956 Egypt

**Keywords:** Orodispersible film tadalafil, Tadalafil tablet, Erectile dysfunction

## Abstract

**Purpose:**

We aimed to compare the efficacy, safety, and compliance of tadalafil 5 mg daily dose in the tablet form versus oral dispersible film (ODF) in men with mild-to-moderate erectile dysfunction (ED).

**Methods:**

One hundred thirty-five randomized patients were equally divided into three groups according to age where each group included forty-five patients. Within each group, 15 patients received oral tadalafil 5 mg, 15 patients received ODF tadalafil 5 mg and 15 patients received a placebo once daily for 1 month. All participants were assessed by the validated Arabic version of the international index of erectile function (ArIIEF-5) at baseline and after 1 month. Also, the efficacy of different forms of tadalafil 5 mg was assessed by responding affirmatively to a questionnaire.

**Results:**

Patients aged > 25 to < 40 years and 40–55 years and > 55 years showed a statistically significant improvement of ArIIEF-5 scores after tadalafil 5 mg tablet and ODF tadalafil 5 mg compared to placebo ODF (23 ± 1.4; 22.7 ± 0.9; 20 ± 0.9; 20.4 ± 1.3; 20.2 ± 1.2; 16.6 ± 1.2; 18.5 ± 1.7; 19.6 ± 1.4; 16.3 ± 1.4; *p* < 0.001, respectively). Three patients (> 25 to < 40 years) who received tadalafil 5 mg tablet showed muscle and back pain. Gastrointestinal (GIT) upset (eight patients) followed by headache (seven patients) were the main side effects reported in patients (40–55 years) who received tadalafil 5 mg tablet. While GIT upset was the main side effect reported in patients (> 55 years) who received tadalafil 5 mg tablet.

**Conclusion:**

ODF tadalafil 5 mg is an effective, tolerable, and safe formulation that can be used in patients with mild-to-moderate ED.

## Introduction

Erectile dysfunction (ED) is a common condition that affects 20–40% of men between the ages of 60 and 69 years and more than 50% of men older than 70 years worldwide [1]. It affects around 20–30% of Egyptian married males [2]. Remarkably, the prevalence is projected to be 80% among patients with type 2 diabetes or metabolic syndrome [2]. Aging, depression, obesity, smoking, obstructive lung diseases, hypertension, dyslipidemia, socioeconomic class, rural residency, quality of life, and COVID-19 infection are all factors contributing to increased incidence and prevalence of ED among the Egyptian population during the past decade [3–5]. The phosphodiesterase type 5 inhibitors (PDE5Is) sildenafil, tadalafil, vardenafil, and avanafil are the first line management of patients diagnosed with ED followed by self-administered intracorporal injection therapy using vasodilator drugs [6]. Moreover, surgical treatment of ED with multicomponent inflatable penile implants was associated with high satisfaction rates [6]. Brock and colleagues (2002) presented their initial data supporting the efficacy and safety of tadalafil [7]. Tadalafil was approved for use in Europe in late (2002), and in November (2003), it was approved by the FDA for use in the United States [7]. Tadalafil’s molecular structure is different than similar structures of sildenafil and vardenafil [8]. Tadalafil is more selective for PDE5 than most other families of PDEIs [8]. Tadalafil had been developed as yellow film-coated tablets (FCTs) intended for oral administration [9]. They are available in several strengths, including 2.5 mg, 5 mg, 10 mg, or 20 mg. However, it is worth noting that this tablet formulation may provide challenges for patients with ED [9].The oral dispersible film (ODF) is a novel type of medication delivery that consists of a small strip of thin polymeric film like a postage stamp in size [10].

The film rapidly disintegrates or dissolves when it is placed on the tongue, allowing for immediate drug release [10]. An ODF can rapidly dissolve and disintegrate within the oral cavity in less than 1 min [11]. The utilization of an ODF has demonstrated enhanced clinical efficacy of medications in comparison to conventional formulations such as tablets or capsules [12]. Enhancements to the taste and the performance of ODFs could be achieved by modifications to various constituents, such as film-forming polymers, active medicinal compounds, plasticizers, flavors, and sweeteners [10, 13]. ODF formulation could be taken without the need for water or chewing, which offered physicians and patients a novel and attractive option for the treatment of ED [14]. Furthermore, ODF formulation could provide a cost-effective alternative to conventional tablet formulation owing to recent developments in ODF manufacturing technology [14]. Thus, we conducted a single-blinded, randomized, placebo-controlled clinical trial to compare the efficacy, safety, and compliance to tadalafil 5 mg daily dose in the tablet form versus ODF in men with mild-to-moderate ED as they were given once daily for 1 month.

## Patients and methods

The study protocol was approved by the local ethical committee of Kasr Alainy Faculty of Medicine. Approval was granted on December 2022 (MS-24-2023) that conforms to Helsinki Declaration 2013 [15]. All participants were recruited from October 2022 to August 2023. Also, they signed a written informed consent after discussing all study procedures, potential risks, and anticipated benefits.

Power of the study and sample size calculator was used for a non-inferiority randomized controlled study, with 0.05 alpha error and power of the study 0.80, 0.1 non-inferiority margin, and 2 enrollment ratios. Accordingly, 135 patients diagnosed with ED were needed to be enrolled and were randomized by simple numbering technique.

### Inclusion criteria

Any married male patient ≥ 25 years old with regular heterosexual intercourse, 2–3 times/week in the previous 6 months prior to enrollment in the study presenting with mild-to-moderate ED was included.

### Exclusion criteria

Patients with diabetes, hypertension, metabolic syndrome, malabsorption syndrome, ischemic heart disease, atherosclerosis, vasculitis, major psychological problems, or Peyronie’s disease were excluded from the current study. Those with post-priapism ED, a history of recent penile and/or urethral surgery or trauma, as well as uncorrected hypogonadism and contraindication to PDE5Is were excluded. Finally, patients who tried PDEIs and experienced side effects were also excluded from the study.

All patients who fulfilled the inclusion criteria were subjected to the following: medical and surgical histories were obtained from the participants. Furthermore, general and local examinations were done. All subjects answered a copy of the validated Arabic version of IIEF-5 (ArIIEF-5) separately at baseline and after 1 month [16]. Morning serum testosterone and prolactin (before 11 AM), total PSA (only in groups including men above 40 Y), glycosylated hemoglobin, random blood sugar, lipid profile were analyzed, and complete urine analysis was performed.

Patients were equally divided into 3 groups according to age where each group included 45 patients. Group A was > 25 to < 40 years, group B was 40 to 55 years, and group C was > 55 years. Within each group, 15 patients received oral tadalafil 5 mg tablet and 15 patients received ODF formulations of tadalafil 5 mg as well as 15 patients received an ODF placebo (hydroxy profile methyl cellulose polymer and starch) for 1 month. Moreover, the efficacy of different forms of tadalafil was assessed by responding affirmatively to the following questions: “Are you satisfied with the effect of treatment on your erections?” and “If yes, has treatment improved your ability to engage in sexual activity?’’ [17]. Finally, all patients were evaluated for the presence of adverse effects such as headache, dizziness, palpitation, gastrointestinal upset, and muscle or back pain on a scale from 1 to 10.

### Statistical analysis

Data analysis was conducted using SPSS version 22nd; Qualitative data were presented by number and percentage, and quantitative data were presented by mean, standard deviation, minimum, and maximum. The Pearson Chi^2^ test was used to compare categorical variables between groups. Quantitative variables were presented in mean, standard deviation, minimum, and maximum. The Kruskal–Wallis test was used to compare quantitative variables between study groups.

## Results

**Fig. 1:**
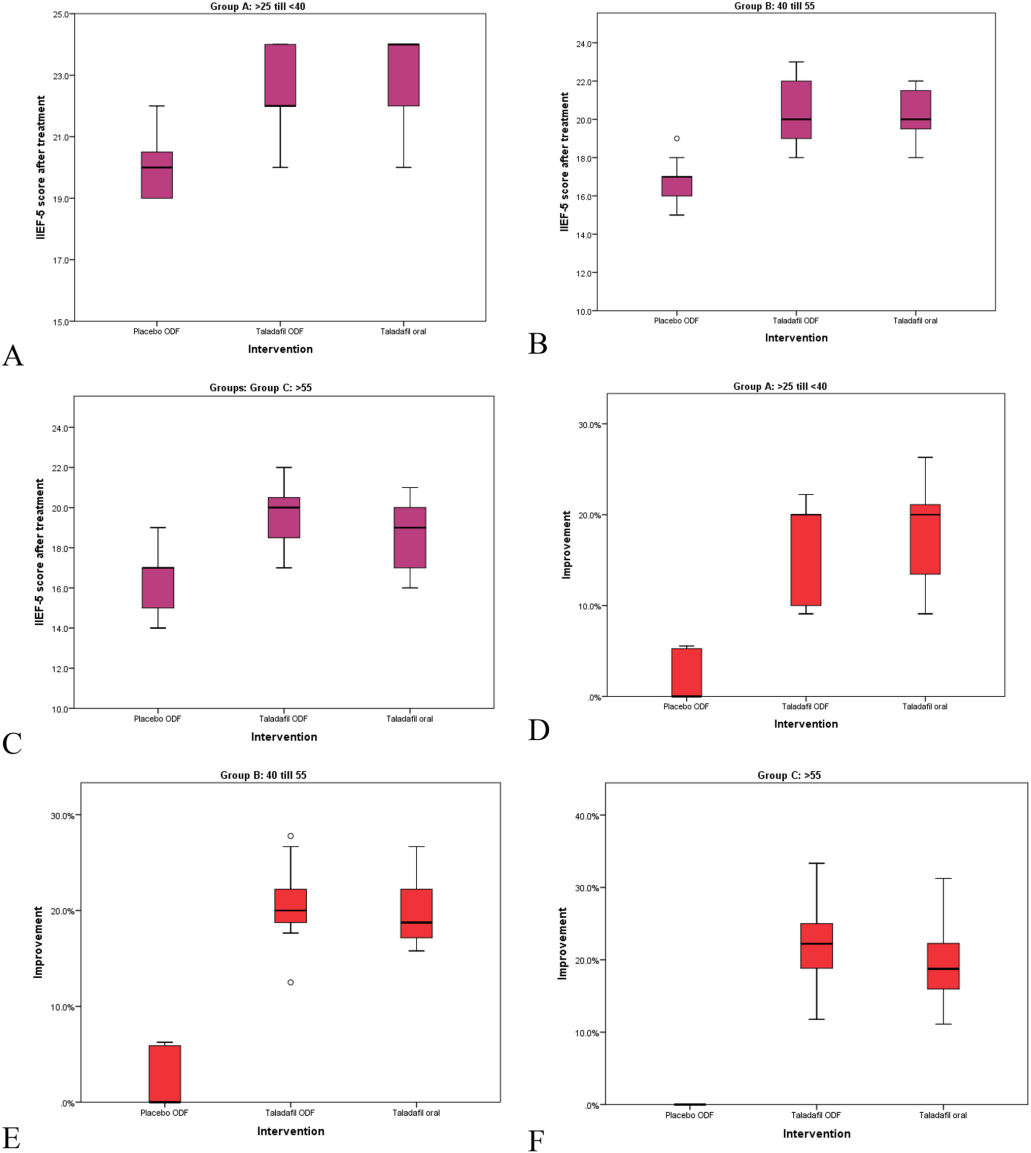
Box plot showing ArIIEF-5 score post-treatment and improvement compared to baseline among age groups according to intervention

Socio-demographic characteristics are presented in Table 1. Groups A, B, and C showed a statistically significant improvement in the ArIIEF-5 scores after treatment with tadalafil 5 mg tablet and ODF tadalafil 5 mg compared to ODF placebo (*p* < 0.001) (Table 2, Fig. 1).

**Table 1:** Sociodemographic characteristics among participants

		Group A > 25 to < 40 years		Group B 40 to 55 years		Group C > 55 years		
		Count	%	Count	%	Count	%	*P* value
Special habits	Ex-smoker	3	6.7%	3	6.7%	5	11.1%	0.466
	Non-smoker	25	55.6%	15	33.3%	18	33.3%	0.039
	Smoker	17	37.8%	27	60.0%	22	46.7%	0.030
Spouse age		27.1 ± 3.7	19–33	43.1 ± 4.7	32–50	48.7 ± 3.4	39–56	< 0.001
Duration of the complaint (months)		7.2 ± 8.9	1–36	13.8 ± 10.8	2–48	52.8 ± 41.2	3–120	< 0.001
Surgical history	Anal fistulectomy	0	0.0%	1	2.2%	0	0%	0.081
	Appendectomy	2	4.4%	3	6.7%	0	0.0%	
	Bil HLO	0	0.0%	3	6.7%	0	0.0%	
	Hemorrhoidectomy	1	2.2%	3	6.7%	0	0.0%	
	Hernioplasty	0	0.0%	1	2.2%	0	0.0%	
	Laryngeal operation	0	0.0%	0	0.0%	1	2.2%	
	Nephrolithotomy	1	2.2%	1	2.2%	0	0.0%	
	Ophthalmology operation	0	0.0%	0	0.0%	1	2.2%	
	Pelvic fracture	0	0.0%	1	2.2%	0	0.0%	
	Piles	0	0.0%	2	4.4%	0	0.0%	
	Pilonidal sinus	0	0.0%	1	2.2%	0	0.0%	
	Rectal prolapse	0	0.0%	1	2.2%	0	0.0%	
	Road traffic accident	0	0.0%	1	2.2%	0	0.0%	
	Testicular biopsy	1	2.2%	0	0.0%	0	0.0%	
	Free	40	88.9%	27	60.0%	43	95.5%

**Table 2:** Comparison between ArIIEF-5 improvement, efficiency, compliance, and safety among participants according to age groups

		Placebo ODF		Tadalafil ODF			Tadalafil tablet				P value
		Mean ± SD	Min–Max	Mean ± SD	Min–Max		Mean ± SD		Min–Max		
Group A (> 25 to < 40 years)	ArIIEF-5 score before treatment	19.6 ± 1.2	18–22	19.5 ± 1.2	17–22		19.5 ± 1.4		17–22		0.99
	ArIIEF-5 score after treatment	20 ± 0.9	19–22	22.7 ± 0.9	20–24		23 ± 1.4		20–24		< 0.001
Group B (40 to 55 years)	ArIIEF-5 score before treatment	16.2 ± 0.9	15–18	16.7 ± 0.9	15–18		17 ± 1.3		15–19		0.171
	ArIIEF-5 score after treatment	16.6 ± 1.2	15–19	20.2 ± 1.2	18–23		20.4 ± 1.3		18–22		< 0.001
Group C (> 55 years)	ArIIEF-5 score before treatment	16.3 ± 1.4	14–19	16.1 ± 1.4	14–19		15.5 ± 1.4		13–18		0.323
	ArIIEF-5 score after treatment	16.3 ± 1.4	14–19	19.6 ± 1.4	17–22		18.5 ± 1.7		16–21		< 0.001
Efficacy											
Group A (> 25 to < 40 years)	Efficient	5	33.3%	15	100%	15		100%		< 0.001	
	Not efficient	10	66.7%	0	0%	0		0%			
Group B (40 to 55 years)	Efficient	6	40%	15	100%	15		100%		0.001	
	Not efficient	9	60%	0	0%	0		0%			
Group C (> 55 years)	Efficient	0	0%	15	100%	15		100%		< 0.001	
	Not efficient	15	100%	0	0%	0		0%			
Compliance											
Group A (> 25 to < 40 years)	Compliant	15	100%	15	100%	15		100.0%		NA	
	Not compliant	0	0%	0	0%	0		0.0%			
Group B (40 to 55 years)	Compliant	15	100%	15	100%	5		33.3%		< 0.001	
	Not compliant	0	0%	0	0%	10		66.7%			
Group C (> 55 years)	Compliant	15	100%	15	100%	9		60.0%		< 0.001	
	Not compliant	0	0%	0	0%	6		40.0%			
Adverse effects											
Group A (> 25 to < 40 years)	Muscle or back pain	0	0%	0	0%	3		20.0%		0.04	
Group B (40 to 55 years)	Headache	0	0%	0	0%	7		46.7%		< 0.001	
	GIT upset	0	0%	0	0%	8		53.3%		< 0.001	
	Muscle or back pain	0	0%	0	0%	4		26.7%		0.012	
Group C (> 55 years)	GIT upset	0	0%	0	0%	6		40.0%		0.001	
	Muscle or back pain	0	0%	0	0%	2		13.3%		0.123	

*N.B ODF* *o*ral dispersible film, *GIT upset* gastrointestinal upset

The efficacy of ODF tadalafil 5 mg and tadalafil 5 mg tablet was markedly higher compared to placebo (*p* < 0.001). Improvement in both formulations was reported in all patients compared to placebo across all groups. Compliance was significantly higher among patients on ODF tadalafil 5 mg compared to tadalafil 5 mg tablet in groups B and C, respectively (*p* < 0.001) (Table 2). Regarding adverse effects, muscle and back pains were significantly higher among patients receiving tadalafil 5 mg tablet compared to other formulations in group A (*p* = 0.040) (Table 2). While headache, gastrointestinal (GIT) upset, and muscle and back pains were significantly reported among patients receiving tadalafil 5 mg tablet compared to other formulations in group B (*p* < 0.001, < 0.001, and 0.012, respectively) (Table 2). Finally, GIT upset was significantly reported among patients receiving tadalafil 5 mg tablet compared to other formulations in group C (*p* < 0.001) (Table 2).

## Discussion

The core findings of the current study were that participants across all groups showed a statistically significant improvement in the ArIIEF-5 scores after tadalafil 5 mg tablet and ODF tadalafil 5 mg compared to ODF placebo. Furthermore, post hoc analysis showed that improvement after tadalafil 5 mg tablet and ODF tadalafil 5 mg was mainly significant compared to placebo, and both formulations were comparable in efficacy. Patients who received ODF tadalafil 5 mg were significantly more compliant to treatment compared to tadalafil 5 mg tablet.

In contrast, Park et al. (2018) stated that safety and tolerability features of the ODF formulation were found to be comparable to those of the film-coated tablet formulation [18]. Furthermore, the same aforementioned study conducted by Park et al. (2018) [18] reported no serious adverse events in the study arms, either in oral tablet tadalafil or ODF tadalafil [18], which could also be seen as contradictory to our findings. In the same context, Cocci et al. (2017) [19] revealed that sildenafil ODF exhibited comparable levels of safety and efficacy to the conventional film-coated tablet [19]. However, the aforementioned study revealed that the ODF formulation elicited greater overall satisfaction among the patients [19], which could be seen as similar to our findings. In the same context, the prevalence of headache in ODF decreased, and the duration and intensity of flushing and nasal congestion were lower in a study conducted by De Toni et al. (2018) [20] that could be seen in agreement with the current findings. Although both formulations demonstrated comparable efficacy in the current study, yet, the ODF tadalafil 5 mg formulation demonstrated lesser side effects as well as better tolerability. Henceforth, the findings of the current study could be postulated that a rapid ODF pre-gastric absorption resulted in a faster onset of action with subsequent enhanced bioavailability and therapeutic outcomes as well as reduced dosing and adverse effects [21]. Furthermore, the safety profiles could be enhanced by lowering toxic metabolites that resulted from hepatic metabolism since the drug was mostly absorbed from buccal mucosa [14]. The main limitation of the current study was the relatively small sample size per group. Also, the inability to measure the serum levels of both formulations to evaluate the pharmacokinetic properties can be added as a limitation.

Finally, exclusion of patients with serious medical comorbidities which might have interfered with the effectiveness of each drug formulation can be regarded as another limitation. However, it should be emphasized that we did not need to monitor any potential side effects from both formulations of tadalafil 5 mg administration especially cardiovascular complications since patients with severe ED and associated serious comorbidities were excluded from the study as mentioned in the previous limitation. Furthermore, it is worth mentioning that proper diagnosis and treatment of ED as well as its risk factors optimize control and management of any associated comorbidity with ED [22]. Eventually, large prospective studies should be conducted to assess the prevalence and severity of adverse events of tadalafil tablets compared to ODF and to assess the difference of tadalafil tablets among patients with organic ED due to medical comorbidities, metabolic and malabsorption syndromes.

## Conclusion

ODF tadalafil 5 mg is an effective, tolerable, and safe drug formulation that can be used regularly or on-demand exactly as tadalafil 5 mg tablets with lesser adverse events and higher compliance rates.

## Data Availability

The data that support the findings of this study are available from the corresponding author upon reasonable request.
